# Staging Systems and Nomograms for Soft Tissue Sarcoma

**DOI:** 10.3390/curroncol30040278

**Published:** 2023-03-26

**Authors:** Maria Danieli, Alessandro Gronchi

**Affiliations:** Department of Surgery, Fondazione IRCCS Istituto Nazionale dei Tumori, Via Giacomo Venezian, 1, 20133 Milan, Italy

**Keywords:** sarcoma, nomogram, prognosis, survival, recurrence

## Abstract

Reliable tools for prognosis prediction are crucially needed by oncologists so they can tailor individual treatments. However, the wide spectrum of histologies and prognostic behaviors of sarcomas challenges their development. In this field, nomograms could definitely better account for their granularity compared to the more widely used AJCC/UICC TNM staging system. Nomograms are predictive tools that incorporate multiple risk factors and return a numerical probability of a clinical event. Since the development of the first nomogram in 2002, several other nomograms have been built, either general, site-specific, histology-specific, or both. Recently, some new “dynamic” nomograms and prognostic tools have been developed, allowing doctors to “recalculate” a patient’s prognosis by taking into account the time since primary surgery, the event history, and the potential time-dependent effect of covariates. Due to these new tools, prognosis prediction is no longer limited to the time of the first computation but can be adapted and recalculated based on the occurrence (or not) of any event as time passes from the first computation. In this review, we aimed to give an overview of the available nomograms for STS and to help clinicians in the process of selecting the best tool for each patient.

## 1. Introduction

Reliable tools for prognosis prediction in cancer patients are used by clinicians to set up adequate management of the disease. The more accurate the prediction, the more specific and tailored the treatment we might propose. Furthermore, these tools might help healthcare professionals address the need for awareness that cancer patients might express, possibly reducing the stress related to the unpredictability of their condition.

Soft tissue sarcomas constitute a heterogeneous family of tumors comprised of >70 histological types arising in nearly any site of the body and with a wide spectrum of prognostic behaviors that goes from indolent entities, such as dermatofibrosarcoma protuberans, to extremely aggressive tumors with a high risk of relapse and death, such as leiomyosarcoma (LMS) or pleomorphic sarcomas.

The American Joint Committee on Cancer/Union for International Cancer Control (AJCC/UICC) TNM staging system, based on tumor size, nodal involvement, and presence of metastases, represents the most widely used tool for prognosis prediction in several cancers. However, as will be later discussed, even the recently updated version (i.e., the 8th edition) carries important limitations in the field of sarcomas. To overcome the limitations of the AJCC/UICC TNM staging system, newer tools for individualized prognosis prediction were developed, i.e., nomograms. The 8th edition of the AJCC manual system endorsed a validated nomogram for retroperitoneal sarcoma that met all 16 AJCC inclusion/exclusion criteria listed by the AJCC Precision Medicine Core to identify reliable and valid prognostic tools [1,2]. Nomograms could definitely better account for the granularity of prognostic entities that characterize the world of sarcomas. In addition, they allow the inclusion of new prognostic markers (such as immunologic, radiologic, radiomic, or genomic markers) that might potentially refine further their predictive ability. In recent years, several nomograms for sarcomas were built and validated, providing experts with a wide choice of prognostic tools.

With this review, we aim to review the recent 8th edition of the AJCC/UICC staging system and give an overview of the available nomograms for STS, helping clinicians in the process of selecting the best tool for each patient.

## 2. American Joint Committee on Cancer/Union for International Cancer Control (AJCC/UICC) TNM Staging System

The 7th edition of the AJCC/UICC TNM staging system (2010) classified STS patients into four stages of disease according to malignancy grade, tumor dimension, tumor depth, lymph node involvement, and distant metastasis [3]. Although it represents the most commonly used tool for prognosis prediction in several cancers, this system has been proven inadequate in soft tissue sarcomas for several reasons [4]. Firstly, it did not take into account histology, which is one of the most important prognostic factors in sarcomas. Although all arising from mesenchymal tissues, STS encompass > 50 different histologic types and all have varying epidemiology, clinical features, biology, responses to therapy, and prognoses. Secondly, the AJCC/UICC TNM staging system did not include site, even though biological behavior, local recurrence risk, and distant metastasis risk are often site-dependent, and STS may arise at nearly any site. Moreover, some authors questioned the inclusion of the N category, which might not be of crucial importance since STS rarely metastasizes to lymph nodes, except for a few types.

For these reasons, a new version of the AJCC/UICC TNM staging system was released in 2017 (8th edition) and includes four different site-specific staging systems for STS: trunk and extremities, retroperitoneum, head and neck, and abdomen and thoracic visceral organs. Other changes were also introduced: 1. in light of the correlation between size and the risk of distant relapse observed by some authors, tumor dimension (T) is considered a 4-tier categorical variable (compared to > vs. ≤5 cmused in the previous edition), [5,6]; 2. given their worse prognosis compared to N0 patients, N1 patients were included in stage IV; 3. depth was no longer considered [5].

Despite these changes, several studies demonstrated the suboptimal performance of the 8th edition of the AJCC/UICC TNM staging system both for sarcomas of the retroperitoneum and the extremities. The prognostic performance of the 8th edition of the AJCC/UICC TNM staging system in RPS and ESTS was evaluated by Cates using data extracted from the Surveillance, Epidemiology, and End Results (SEER) database and by Fisher et al. using the National Cancer Database (NCDB) [7,8,9].

For RPS, both authors found that size was a weaker prognostic factor compared with grade, histology, and incomplete resection [7,8]. Indeed, size seems to have a peculiar effect on prognosis, with an unfavorable effect that lessens progressively for tumors up to 25–30 cm and becomes protective thereafter [2,10]. Clearly, the current AJCC 8th edition staging system is not structured to capture this relationship. A similar nonlinear effect was registered in ESTS, again raising questions about the clinical significance of the new 4-tiered T staging system [9]. With regards to ESTS, two further issues were highlighted. First, both studies showed that patients with metastatic nodal disease had a worse prognosis compared with patients with locally advanced disease but a better survival outcome compared with patients with distant metastasis and should therefore not be merged with these latter. Second, depth seems to significantly contribute to prognosis determination for intermediate- and high-grade tumors and should not have been removed.

In light of these observations, Cates proposed two new staging systems (Vanderbilt staging systems), one for RPS using size (≤10 cm, >10 and ≤15, >15), histologic grade, histologic type, and presence of distant non-nodal metastasis, and one for ESTS based on histologic grade, tumor size, and depth. The Vanderbilt staging systems were compared to the 8th and 7th editions of the AJCC Cancer Staging Manual. At the internal validation, Vanderbilt staging systems performed better than both editions of the AJCC Cancer Staging Manual, and the 8th edition showed a worse discriminative ability compared to the 7th in RPS, while it was not better than the 7th in ESTS.

## 3. Nomograms

The prognostication ability of the TNM staging system remains limited by the disproportioned importance attributed to anatomic variables and the penalization of pathological or patient-related variables. Furthermore, in the AJCC/UICC TNM staging system, patients are forced into a limited number of stages that are supposed to reflect their risk of recurrence or relapse, even though this risk would be better expressed as a continuous one.

Nomograms are predictive tools that incorporate multiple risk factors and return a numerical probability of a clinical event. The computation might be performed through a simple graphical representation or, when available, through web-based calculators. Of note, risk calculation through nomograms accounts for the mutual influence of each prognostic variable on the others. In this way, the relative weight of every variable is not static, as in TNM, but changes according to the case, with the resulting computation giving a more accurate estimation of the real risk.

In addition, nomograms find an application in the field of clinical research and were used to perform risk stratification of patients [10,11].

Still, some limitations do exist and are mainly related to the fact that the prognosis can change over time, thus making the prediction less reliable as one moves away from the time it was computed, usually after surgery. In fact, the risk of recurrence or of tumor-related death can change over time, generally being higher in the first years of follow-up and decreasing as time goes by after surgery. Furthermore, the occurrence of an event or the lack of events does affect prognosis, e.g., DM affects prognosis unfavorably, and vice versa, the lack of events affects prognosis favorably. Moreover, some prognostic factors might have a time-dependent effect (i.e., their weight on the prognosis might increase or decrease over time during follow-up). For these reasons, some new “dynamic” nomograms and prognostic tools were developed [12,13,14], aiming to “recalculate” a patient’s prognosis factoring the time elapsed since primary surgery, the event history, and the possible time-dependent effect of covariates. These new tools might help clinicians develop tailored FU policies and refine their approach to treating patients who recur.

Nomograms can be site-specific, histology-specific, or both. According to this classification, we aimed to give an overview of the available nomograms for STS.

### 3.1. General Postoperative Nomograms for STS

The first nomogram for patients with STS was built by Kattan et al. in 2002 at Memorial Sloane Kettering Cancer Center (MSKCC) [15] (Table 1). This model—referred to as the MSKCC Sarcoma Nomogram (MSKSN)—computes two separate predictions for high-grade and low-grade sarcomas to estimate the probability of sarcoma-related death within 12 years of his initial surgery. In the online version (https://www.mskcc.org/nomograms/sarcoma) (accessed on 9 March 2023), sarcoma-specific death predictions are also computed at 4 and 8 years. Nomogram predictor variables include age at diagnosis, tumor size (<5, 5 to 10, or >10 cm), histologic grade (high or low), histologic type (seven categories), depth (superficial or deep), and site (upper extremity, lower extremity, visceral, thoracic or trunk, retro-intraabdominal, or head or neck). Among the strengths of the model are the large development cohort (2163 patients) and the extensive external validation that always showed good calibration and discrimination [16,17,18,19,20,21,22,23,24]. Indeed, it was proven to be a valid prognostic tool in patients with truncal and extremity sarcomas [16] as well as head and neck sarcomas [23]. With regard to RPS and its applicability on these patients, initial questions were raised since the developing cohort included only a minority of patients with RPS (13%), and a first validation on a cohort of RPS patients only treated at MSKCC showed a relatively low H-index (0.60) [24]. However, a recent validation conducted on an external cohort of RPS demonstrated good prognostic ability also in these sarcomas, with a CI of 0.71 comparable to the C-indices of 0.77 and 0.76 from the original development and validation cohorts [25]. Wong et al. validated the MSKSN nomogram on a cohort of patients with RPS operated on in a tertiary center in Singapore, proving its applicability to Asian patients with retroperitoneal tumors [26]. A previous validation on a larger Asian cohort of STS sites suggested that the MSKSN maintained good discrimination but overpredicted survival among patients with lower survival probabilities in this population [21].

The MSKSN was applied to a series of pediatric patients with non-rhabdomyosarcoma adult-type soft tissue sarcomas with the aim of exploring differences and similarities in outcomes and in the effect of clinical-pathological prognostic factors between the adult and pediatric populations [27]. According to this study, the prognostic variables adopted in the MSKSN for predicting survival in adult STS are also relevant for pediatric patients with “adult-type” STS. However, the actual 10-year mortality of the series was significantly higher than predicted, possibly due to the stronger negative prognostic effect of tumor size and tumor depth. Furthermore, the age-related mortality pattern predicted by the nomogram can be extrapolated below the age of 16 (which is the minimum age in the nomogram).

Szkandera et al. demonstrated that the addition of the preoperative C-reactive protein level, the fibrinogen level, and the lymphocyte/monocyte (L/M) ratio (all significant prognostic factors in multivariate analysis) to the MSKSN model improved the Harrell C-index from 0.74 to 0.77, from 0.75 to 0.78, and from 0.74 to 0.78, respectively [19,20,28].

Despite its extensive external validation, the MSKCC model presents several limitations. First, it has never been updated since its development. In fact, over the past few years, the changes in the quality of the treatments delivered might have led to a significant improvement in the prognosis of these patients. In addition, advances in immunohistochemistry and molecular pathology led to an update of the WHO histologic classification, and some histological entities no longer exist (e.g., “malignant fibrous histiocytoma”) or have been reclassified. Second, tumor size is managed as a categorical variable. Hence, the system cannot capture outcome differences between patients with identical characteristics except for tumor dimensions when their sizes fall in the same interval (e.g., 5 cm vs. 10 cm). Third, histologic grade was classified as low or high, while the 3-tier FNCLCC (Fédération Nationale des Centres de Lutte Contre Le Cancer) grading system has been proven to correlate better with patients prognoses compared with a 2-tier system [24,29]. Finally, site- and/or histology-specific prognostic tools have been proven to have better stratification ability and should therefore be preferred whenever available. In the absence of a more specific nomogram (e.g., head and neck STS), the MSKCC nomograms continue to be a useful prognostic tool for all histologies and sites.

### 3.2. Nomograms for Extremity STS

Approximately half of all sarcomas arise in the extremities. Sarcomas exhibit a wide range of behaviors due to their high histologic variability (>70 histologic types). More frequently, these patients die from the metastatic spread of the disease. A local recurrence can be managed with limb-sparing re-resection or amputation, except when occurring at critical sites (e.g., girdles invading the pelvis or chest), where it may lead to death due to uncontrollable locally advanced disease [30].

Nomograms for patients with extremity STS (eSTS) are shown in Table 1.

The first nomogram specific for eSTS was built by Mariani et al. at the Istituto Nazionale dei Tumori (INT) in Milan during the process of external validation of the MSKCC model by substituting the 2-tier grading system used in the MSKCC model with the 3-tier grading system of the FNCLCC [16]. As derived from the MSKCC model, it predicts 10-year-old sarcoma-specific death and has maintained its limitations. The nomogram underwent internal and external validation, showing good calibration and discrimination with a C-index of 0.71 for 4-, 8-, and 12-year disease-specific survival, comparable to the C-indices of 0.77 and 0.76 from the original MSKCC development and validation studies [31].

In 2012, a nomogram to predict the 3- and 5-year risk of LR after limb sparing surgery in patients with primary eSTS who did not undergo (neo)adjuvant radiotherapy or chemotherapy was developed by Cahlon et al. from the MSKCC [32]. Among the limitations of this model are the use of grades classified according to a 2-tiered high- and low-grade system, the use of a dichotomic histological classification (WDLPS/Atypical Lipomatous Tumor versus others, where “others” includes histologic subtypes with significantly different local outcomes), and the use of size and age as categorical variables. In addition, the nomogram cannot be used in the case of the administration of perioperative treatments, which significantly restricts its applicability given their increasing use over the last two decades [30]. Lastly, one should keep in mind that the direct correlation between the local control of the disease and the final oncological outcome in patients with eSTS remains controversial [33]. Indeed, this model is the only available prognostic tool to predict the local outcome after surgical resection in patients with extremity WDLPS, while it does not really help personalize the prediction of local risk for patients affected by all other eSTS types and grades.

**Figure 1:**
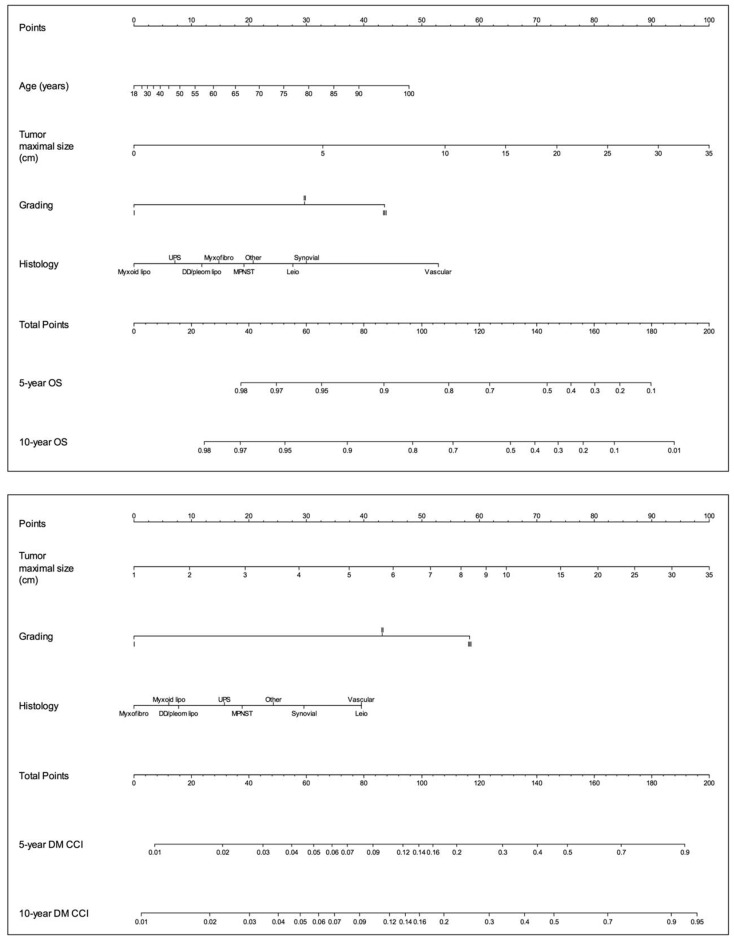
These nomograms allow for the calculation of the 5-year and 10-year probability of (**Top**) overall survival (OS) and (**Bottom**) the crude cumulative incidence (CCI) of developing distant metastases after surgical resection of a primary soft tissue sarcoma of the extremities on the basis of patient-related and tumor-related covariates. The user should locate the values of specific covariates, draw a line up to the point axis to establish the score associated with each covariate, sum the score of each covariate, and locate the total score on the total point axis. By drawing a straight line down to the OS/distant metastases axis, the user would then obtain the probability. DD/pleom lipo indicates dedifferentiated/pleomorphic liposarcoma; Leio, leiomyosarcoma; MPNST, malignant peripheral nerve sheath tumor; Myxofibro, myxofibrosarcoma; Myxoid lipo, myxoid liposarcoma; UPS, undifferentiated pleomorphic sarcoma. Reprinted with permission from Elsevier from [34] (License number 5478680598871).

In 2016, the collaboration of INT, Mount Sinai Hospital (Toronto, Ontario, Canada), Royal Marsden Hospital NHS Foundation Trust (London, UK), and Institut Gustave Roussy (Villejuif, France) led to the development of two multicentric, externally validated nomograms to predict OS and metastatic risk in patients surgically treated for primary eSTS at 5 and 10 years after surgery (Figure 1) [34]. Since it includes age and size as continuous variables (and both demonstrated a nonlinear prognostic effect), grading as a 3-tier system, and an updated histologic classification with nine categories, this model overcomes the limitations of the previous models. Patients with macroscopically incomplete surgical resection and patients with WD liposarcoma were excluded. The Italian series (1452 patients) served as the development cohort, whereas the three other series served as independent external validation cohorts (420 patients for the French validation cohort, 1436 patients for the Canadian validation cohort, and 444 patients for the UK validation cohort). In both validation processes, it showed good calibration and discrimination, and in the following years, it underwent further external validations. These nomograms are available on Sarculator [35], a free app for smartphones and tablets that also includes five other prognostic tools for STS. Squires et al. validated the Sarculator for eSTS on a series of 1326 patients from nine high-volume US institutions in 2022 with excellent results [31], while Voss et al. validated the OS nomogram on 9738 patients from the National Cancer Data Base (NCBD) and estimated concordance indices based on patient and tumor characteristics [36]. The overall CI was 0.726, with variable CIs among different histologic subtypes. The worse performance was registered for liposarcoma (CI = 0.679), MPNST (CI = 0.656), vascular (CI = 0.696), and synovial (CI = 0.691), while the best was registered for leiomyosarcoma (CI = 0.745). The pattern of failed prediction was unique for each histology (e.g., MPNST survival is globally overestimated, while liposarcoma survival tends to be underestimated for lower survival groups). Furthermore, Sarculator was proven to work optimally for younger patients with earlier-stage tumors treated at reference centers, while it overestimated OS for those who were elderly and had two or more comorbidities. Of note, this nomogram is the only externally validated instrument available to date to predict the risk of DM in patients with primary resected extremity STS.

In addition to prognostication, the Sarculator was shown to be a very important predictor of anthracycline plus ifosfamide (AI) chemotherapy efficacy in high-risk STS of the extremities and trunk wall. Indeed, a study analyzing data from ISG-STS 1001—a randomized study that tested AI chemotherapy versus histology-tailored chemotherapy in STS—showed that neoadjuvant AI benefits patients with a Sarculator 10-yr predicted OS < 60%, while this was not true for patients with a lesser risk [10,37]. The use of nomograms to select treatments is now expanding and will be included in the studies to come.

In 2017, van Praag et al. used an international multicentric cohort to develop a model (not a nomogram) that predicts the cumulative incidence of LR and OS for patients with high-grade eSTS [38]. In contrast to Cahlon et al.’s MSKCC nomogram, the PERSARC model is also applicable to patients who have received medical treatments, making it the only prognostic tool capable of predicting the local outcome after surgical resection in patients with eSTS who received (neo)adjuvant ChT or RT.

In 2018, Rueten-Budde et al., expanding the cohort of PERSARC, developed the first dynamic model to predict 5-year OS during the first 5 years of FU in patients operated on for primary high-grade eSTS [39]. Variables included were age, sex, histology, size, preoperative RT and ChT, margins, and depth. Interestingly, the effect of negative margins becomes gradually weaker the longer that patients remain alive during follow-up. A time-varying effect was also registered for the histologic subtype, although histological classification with only five subgroups is suboptimal. The time effect on grading was not tested since all pts were -rade. PERSARC was also successfully externally validated [14].

More recently, INT guided an international multicentric collaboration to develop and externally validate a dynamic nomogram (available on the Sarculator app) that predicts 5-year OS during the first 3 years of follow-up in pts operated on for primary eSTS [12]. It is worth noting that this nomogram was developed with the largest series of patients (3740 patients) from four referral centers and was externally validated on a multicentric series of 893 patients operated at seven European referral centers. The variables selected were: age at surgery; tumor size and its interaction with landmark time (i.e., time at primary surgery); grading and its interaction with landmark time; histology; and both LR and DM (as first event) indicator variables. The nomogram showed good calibration and discrimination. Harrell indexes at different landmark times were between 0.776 (0.761–0.790) and 0.845 (0.823–0.862) in the development series and between 0.675 (0.643–0.704) and 0.810 (0.775–0.844) in the validation series. In this model, the effect of tumor size and grade becomes gradually weaker the longer that patients remain alive during follow-up. Compared to PERSARC, this nomogram is applicable to both high- and low-grade eSTS and includes nine histologic subtypes (versus only five in PERSARC). However, it shows some limitations: first, it does not factor in the effect of a second event, and second, it was not validated on grade I patients.

**Table 1:** General and extremity-specific nomograms for patients with STS.

		Development Series Characteristics			Nomogram Details			Internal Validation	External Validation	
	Study	Selection Criteria	Timeframe	No. of Centers	Predicted Outcomes	No. of Patients	Nomogram’s Covariates (a)	Concordance Index	Yes/No	Concordance Index
General postoperative nomograms	Kattan [15] 2002	Primary, surgically treated, any site but skin	1982–2000	1	12-y low-grade SSD, 12-y high-grade SSD	2163	Histology (7 categories), size (3 categories), age (continuous), site (6 categories), depth (superficial vs. deep)	0.77	Yes	0.64–0.76 (b)
Nomograms specific for extremity STS	Mariani [16] 2005	Primary completely resected extremity STS	1980–2000	1	10-y SSD	642	Grade (3 tiers), histology, age (continuous), size (3 categories), depth (superficial vs. deep), site (lower vs. upper)	0.76	No	
	Cahlon [32] 2012	Primary extremity STS treated with limb-sparing surgery without adjuvant therapy	1982–2006	1	3-y and 5-y LRrate	684	Histology (WDLPS vs. others), surgical resection margin (negative vs. close/positive), grade (low vs. high), age (dichotomic, cutoff at 50 y), size (dichotomic, cutoff at 5 cm)	0.73	No	
	Callegaro [34] 2016	Primary extremity STS treated with surgery	1994–2013	1	5-y and 10-y OS	1452	Size (continuous), histology (9 categories), age (continuous), grading (FNCLCC)	0.77	Yes	0.70–0.77 (c)
					5-y and 10-y DM	1452	Size (continuous), grading (FNCLCC), histology (9 categories)	0.76	Yes	0.65–0.75 (c)
	Van Praag [38] 2017	Primary high-grade extremity STS surgically treated with curative intent	2000–2014	5	3-y, 5- and 10-y cumulative incidence of LR	766	Margin status (0 vs. 0.1–2 mm vs. >2 mm), RT (no, neoadjuvant, adjuvant), size (continuous), age (continuous), depth (deep vs. superficial vs. both), histology (5 categories)	0.68	No	
					3-y, 5- and 10-y OS	766	Age (continuous), size (continuous), RT (no, neoadjuvant, adjuvant), margin status (0 vs. 0.1–2 mm vs. >2 mm, depth (deep vs. superficial vs. both), histology (5 categories)	0.70	No	
	Rueten-Budde [39] 2018	Primary high-grade extremity STS surgically treated with curative intent	2000–2014	14	5-y dynamic OS	2232	Baseline variables: age (continuous), size (continuous), depth (deep vs. superficial), histology (7 categories). Radiotherapy (no vs. neoadjuvant vs. adjuvant), margins (R0 vs. R1-2) Time-dependent variables: LR and DM	0.78	Yes	0.83 (d)
	Callegaro [12] 2019	Primary extremity STS treated with surgery	1994–2013	4	5-y dynamic OS	3740	Age, size and its interaction with landmark time, grading (FNCLCC) and its interaction with landmark time, histology, LR and DM (as first event)	0.78–0.84	Yes	0.67–0.81 (e)

Abbreviations: FNCLCC, Federation Francaise des Centres de Lutte Contre le Cancer; DM, distant metastasis; LR, local recurrence; NA, not applicable; OS, overall survival; SSD, sarcoma-specific death; STS, soft tissue sarcoma; WDLPS, well-differentiated liposarcoma. (a) The variables are listed according to their nomogram score range, which reflects their relative effect on the predicted outcome, on a decreasing basis (the first variable exerts the strongest influence on the predicted outcome). For non-nomogram prediction models, variables are listed according to the p value at multivariable analysis on an increasing basis or according to time dependence for dynamic models. (b) External validations were performed on 929 patients with primary STS from the University of California at Los Angeles (UCLA) (Harrell C-index, 0.76) [17]; 642 patients with primary extremity STS from the National Cancer Institute in Milan, Italy (Harrell C-index, 0.75) 18; 238 high-risk patients with primary extremity STS who were treated with neoadjuvant high-dose ifosfamide-based chemoradiotherapy within the context of a phase 2 prospective trial at UCLA (Harrell C-index, 0.77) [18]; 9237 patients from the Surveillance, Epidemiology, and End Results (SEER) database of the National Cancer Institute (Harrell C-index, 0.74) [22]; 187 patients with primary head and neck STS who were treated at Memorial Sloan Kettering Cancer Center (Harrell C-index, 0.78) [23]; 304 patients with STS who were undergoing surgery at the Medical University of Graz in Graz, Austria (Harrell C-index, 0.74) [19]; 294 patients with STS who were undergoing surgery at the Medical University of Graz (Harrell C-index, 0.747) 26; 340 patients with STS who were undergoing surgery at the Medical University of Graz (Harrell C-index, 0.74) [20]; 399 patients with STS who were undergoing surgery at the National Cancer Centre Singapore (Harrell C-index, 0.71) [21]; 502 patients who underwent resection of primary RPS at nine high-volume academic US institutions (data from the US Sarcoma Collaborative database) (Harrell C-index, 0.71 (95%CI: 0.67–0.75) [25]; and 109 patients who underwent complete resection for primary retroperitoneal sarcoma at the National Cancer Centre Singapore (Harrell C-index, 0.72.) [26]. (c) External validations were performed on 1436 patients from Mount Sinai Hospital in Toronto, Ontario, Canada (Harrell C-index, 0.775 [95% CI, 0.754–0.796] for OS and 0.744 [0.720–0.768] for DM); 444 patients from Royal Marsden Hospital NHS Foundation Trust in London, United Kingdom (Harrell C-index, 0.762 [0.720–0.806] for OS and 0.749 [0.707–0.791] for DM); 420 patients from Institute Gustave Roussy in Villejuif, France (Harrell C-index 0.698 [0.638–0.754] for OS and 0.652 [0.605–0.699] for DM); 1326 patients underwent resection of primary extremity STS at nine high-volume academic US institutions (data from the US Sarcoma Collaborative database) (Harrell C-index 0.73 [CI 0.70–0.75] for OS and 0.72 [CI 0.69–0.75] for DM; and 9738 patients from the National Cancer Data Base with resected stage I–III primary extremity or trunk sarcoma (Harrel C-index 0.726 for OS, DM was not tested). (d) External validations were performed on 1111 patients with extremity STS treated at the Istituto Nazionale dei Tumori, Milan. (e) External validation was performed on 893 patients operated on between 2000 and 2016 at 7 other European referral centers [12].

### 3.3. Nomograms for RPS

Sarcomas arising in the retroperitoneum represent approximately one fifth of all STS. Although virtually any STS may arise at this site, four main histological types account for nearly 90% of tumors in the retroperitoneum, each showing a peculiar pattern of recurrence [40]. Well-differentiated liposarcomas (WDLPS) grow slowly over time and virtually never metastasize, but they have a strong tendency to relapse locally, which mainly affects survival [40,41,42,43]. Dedifferentiated liposarcomas (DDLPS) instead have a more aggressive behavior, showing a higher risk of both LR and DM and a shorter time interval to recurrence [40,41,42,44]. Within this group, some differences in recurrence pattern can be found according to the malignancy grade of the dedifferentiated component: while in G2 DDLPS OS is determined by the risk of LR, similarly to WDLPS, but with a shorter disease-free interval and a lower OS, in G3 DDLPS OS is both determined by LR and DM [41]. Leiomyosarcomas (LMS) show very low rates of LR but the highest rate of DM, which significantly affects survival. Still, OS is better than G3 DDLPS, possibly thanks to the availability of several potentially active drugs in advanced leiomyosarcoma. Solitary fibrous tumors (SFT) display an indolent course, with few LR and DM, although aggressive variants do exist and represent around 10% of SFT [40,41,45].

Together with histology, tumor size, grade, and multifocality are the tumor related factors significantly affecting the risk of recurrence [24,42,43,46,47]. The quality of surgery is the most important treatment-related factor, with macroscopic complete resection being a critical determinant of outcome.

Nomograms for RPS are shown in Table 2.

The first two nomograms for RPS were developed in 2010 by Anaya et al. [48] and Ardoino et al. [10]. Both presented limitations, that were overcome by the later, developed nomograms and are no longer used.

In 2013, Gronchi et al. used the merged data of three referral centers (INT, Milan, Italy; the University of Texas MD Anderson Cancer Center, Houston, TX; and the University of California, Los Angeles, Los Angeles, CA) to develop a nomogram to predict OS after curative intent surgery for RPS and DFS after macroscopically complete surgical resection [2]. Data from the Institut Goustave-Roussy, Villejuif, France, were used as a validation set. Variables included in the nomogram are listed in Table 2. The nomogram is available on the Sarculator app or website [35].

In 2016, Tan et al. used the MSKCC cohort of patients surgically treated for RPS to develop 3 separate nomograms to predict disease-specific death, LR, and DM incidence at 10 years.

While age was included only in the nomogram by Gronchi et al. (as a continuous variable), size was included in both models, as a categorical (less than 10 cm, 10 to 20 cm, and 20 cm or greater) in the model by Tan et al., and as a continuous variable in the model by Gronchi et al. As previously in the nomogram by Ardoino et al. [10], in the Sarculator nomogram, tumor size and the patient’s age showed an interesting nonlinear prognostic pattern. In particular, both increasing patient age and tumor size tended to worsen the prognosis; however, these trends were not apparent for patients younger than 50 years or for tumor sizes greater than 30 cm. In the nomogram by Tan et al. for DM, greater dimensions are associated with lower risk compared with intermediate dimensions.

Although grading has been extensively proven to provide additional prognostic information beyond that derived from histology [40], Tan et al. only distinguish high- and low-grade LMS, while Sarculator includes a 3-tiered grading system. Both include seven histological categories and separate liposarcomas according to differentiation grade.

The extent of resection (R0/R1 vs. R2) is also included in both models to compute survival, while the number of organs resected (<3 vs. ≥3, both in the DSD and in the LR calculation) and multifocality are exclusively factored in the nomogram by Tan et al. and in the Sarculator, respectively.

To the best of our knowledge, only the nomogram by Gronchi et al. has received extensive external validation [25,26,50,51], including validation on a cohort of Asian patients operated on at a tertiary center [26] and validation on a large cohort of 631 patients from a multicentric cohort of the TARPSWG [50], always showing good calibration and discrimination (CI for OS 0.63–0.73, and CI for DFS 0.65–0.73 compared with 0.74 and 0.71 in the development cohort, respectively).

**Figure 2:**
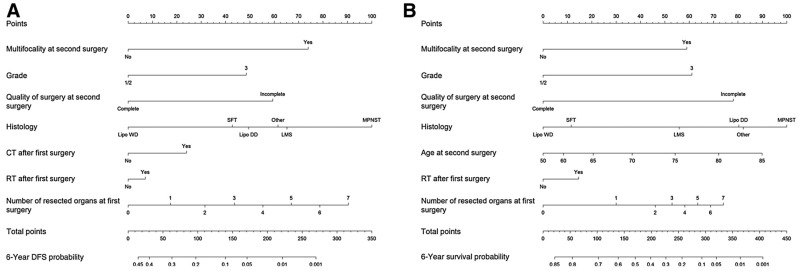
Nomograms to predict 6-year DFS (**A**) and OS (**B**) after second surgery in patients with recurrent RPS. Instructions: each nomogram provides a method of calculating the 6-year DFS or OS probability on the basis of a patient’s combination of covariates. For instance, in the DFS nomogram, locate the patient-specific multifocality at second surgery and draw a line straight up to the “Points” axis to determine the associated score. Repeat for the other nomogram variables, sum the scores, and locate the total score on the “Total Points” axis. Draw a line straight down to the “6-year DFS Probability” axis to obtain the probability. CT, chemotherapy; DFS, disease-free survival; Lipo DD, dedifferentiated liposarcoma; Lipo WD, well-differentiated liposarcoma; LMS, leiomyosarcoma; MPNST, malignant peripheral nerve sheath tumor; OS, overall survival; RT, radiotherapy; SFT, solitary fibrous tumor; UPS, undifferentiated pleomorphic sarcoma. Reprinted from [49] (no permissions needed for coauthors).

In 2019, the TARPSWG collaboration was able to build a nomogram to predict 6-yr DFS and OS in pts who underwent surgery for locally recurrent RPS (Figure 2) [49]. Of note, this is the largest study on locally recurrent retroperitoneal sarcoma and the only available nomogram to predict the outcome after a surgically treated recurrence for RPS. Variables included in nomograms are shown in Table 2.

Of note, this nomogram is not applicable to unresectable cases, which account for almost half of the patients with recurrent RPS. The decision to offer surgery to patients with LR should always be validated by a multidisciplinary tumor board, aiming at balancing the risks related to the planned surgical procedure with the expected oncologic outcomes [52]. In this setting, this tool might support this decision by identifying, among patients eligible for surgery, those who have potentially more favorable outcomes.

In 2021, Callegaro et al. collaborated to develop and externally validate the first dynamic nomogram for RPS [13]. The nomogram was built on a multi-institutional cohort of 1357 patients (the largest development cohort of primary nonmetastatic RPS) and allows to estimate 5-year OS and DFS probability at different time points throughout the first 5 years of follow-up in patients who received surgery for primary (non-recurrent) non-metastatic RPS.

These instruments might help healthcare professionals tailor the follow-up schedule to the actual, updated, and individualized risk, decreasing the intensity of FU in patients at low risk of disease recurrence/death. Of note, the OS dynamic nomogram also accounts for non-tumor-related causes of death, giving a more comprehensive and realistic picture of patients’ prognosis.

Of note, dynamic nomograms find a different application compared to static postoperative nomograms and should be used by experts as complementary tools. In fact, dynamic nomograms can be used anytime during follow-up, either in the absence of events (OS and DFS nomograms) or at the occurrence of an event but before the delivery of any treatment (OS nomogram). Since prognosis computation is performed regardless if the tumor is resectable or not and regardless of the nature of the surgery eventually performed, in the event of a recurrence, the OS dynamic nomogram can guide the decision-making process to select the best treatment. In contrast, the static nomograms for recurrent patients can only be applied after the occurrence of the local relapse and after its surgical treatment, since the completeness of the resection at the time of recurrence is one of the prognostic variables. Of note, once a LR has occurred, DFS can only be estimated by a static nomogram because the dynamic DFS nomogram can only be used in patients with uneventful follow-up. In addition, only static nomograms are able to factor in some important prognostic variables, such as tumor multifocality and the number of organs resected at primary surgery.

Nonetheless, all the available nomograms for RPS still share some important limitations. First, none of them can be used in the preoperative setting because some of the variables included are not available before surgery. Second, none of them is designed or applicable to patients with metastatic or unresectable disease at diagnosis. Only the dynamic nomogram developed by Callegaro et al. can be applied to patients who develop metastases after surgery for a resectable localized RPS [13]. Third, they might become invalid with the possible increase in the use of preoperative treatments in light of the recent evidence showing a potential benefit in terms of LR in patients with WD and low-grade DD LPS and low-grade differentiated liposarcoma pretreated with RT [53].

With the hypothesis that neoadjuvant RT might interfere with the applicability of existing RPS-specific nomograms [2,10,24,48], Ng et al. tested their predictive accuracy on a prospective series of 840 patients managed at Princess Margaret Cancer Centre/Mount Sinai Hospital, Toronto, where more than 80% of patients surgically treated for RPS received preoperative radiation, compared to 8–32% of patients who received (neo)adjuvant RT in the development cohorts [54]. The authors found the performance of available nomograms was worse in this cohort; in particular, the concordance index for LR predicted with the MSKCC nomogram [24] was very poor (0.61) and even poorer if the analysis was conducted only on patients who received preoperative RT (0.59) compared to a very good CI (0.89) on the “no preop RT cohort”. Similarly, a worse performance was registered when the nomogram by Gronchi et al. [2] was applied to the “no preop RT cohort” compared to the “preop cohort”, both to estimate OS and DFS. These results, possibly supporting the role of preoperative radiation in lowering the risk of LR following resection for primary RPS, appear to question the applicability of some components of existing prognostic nomograms for primary RPS and to ask for newer tools that factor perioperative treatments into the risk computation.

### 3.4. Histology-Specific Nomograms

#### 3.4.1. Liposarcoma

Liposarcoma (LS) is the most common soft tissue sarcoma (STS), accounting for 20% of all sarcomas in adults. There are four main histological subtypes of liposarcoma, with dedifferentiated liposarcoma and well-differentiated liposarcoma arising mainly in the retroperitoneum and myxoid liposarcoma and round cell liposarcoma arising mainly in the extremities. Histology, site, and differentiation grade are the most important determinants of prognosis.

In 2016, Dalal et al. from the MSKCC developed a nomogram to predict 5- and 12-year disease-specific survival in patients surgically treated for liposarcoma, assuming that the patient does not die of another cause first [55] (Table 3). Grade was not included in the model since histology was supposed to enclose this information, as supported by the higher concordance index obtained when histologic subtype is included in the model compared to a 2-tiered grading system (CI = 0.817). Only the round cell variant was further divided into two categories according to the percentage of round cells (between 5 and 25% and more than 25%). Nonetheless, it is well known that in DD-LPS of the retroperitoneum, low-grade (G2) disease has different outcomes from high-grade (G3) disease, and grade might still add some prognostic information to those given by histology [24,42,46]. Of note, similarly to general RPS nomograms, a limitation of this liposarcoma-specific nomogram is that it does not factor in the use of perioperative treatments. The nomogram underwent internal validation and showed better discriminative ability compared to the MSKCC general nomogram applied to the same cohort (CI = 0.827 vs. 0.776).

#### 3.4.2. Synovial Sarcoma

Accounting for approximately 8–10% of all soft tissue sarcomas, synovial sarcoma typically occurs in adolescents and young adults (mean age of 39 at diagnosis) and shows aggressive behavior with a strong tendency to metastatic spread. The translocation between chromosomes X and 18 (leading to the expression of several different SS18:SSX fusion proteins) is present in more than 95% of patients and is therefore considered pathognomonic. Three main histologic variants do exist: monophasic SS, biphasic, and poorly differentiated. Validated negative prognostic factors are older patient age at diagnosis, male sex, larger tumor size, positive surgical margins, advanced stage, and site (with a worse outcome for tumors arising from anatomic sites other than the extremities). Conversely, the prognostic role of histological subtype and fusion protein status is less clear [56].

In 2008, Canter et al. from the MSKCC group developed a nomogram based on preoperative variables to calculate 3- and 5-year disease-specific survival (DSS) in surgical patients with synovial sarcoma who did not receive preoperative ChT. When applied to a group of patients who pretreated with anthracycline-ifosfamide, the observed DSS for the first 3 years of follow-up was better than expected, supporting a possible role of chemotherapy in survival in these patients [57]. Only tumor size and site were independent negative prognostic factors in the multivariable analysis of disease-specific death.

#### 3.4.3. Rhabdomyosarcoma

Rhabdomyosarcoma (RMS) is the most common soft tissue sarcoma in children, represents approximately 5% of all childhood cancers, and can arise in nearly any site. According to the WHO classification, there are four main histologic subtypes with different biological behavior and prognosis: embryonal, alveolar, pleomorphic, and spindle cell/sclerosing. In addition, RMS can be classified according to the presence or absence of the PAX-FOXO1 fusion protein in fusion positive or fusion negative RMS. Fusion-negative tumors, embryonal subtype, age 3–10, and head and/or neck and genitourinary sites are all favorable prognostic factors, while age > 10 years, tumors arising in the trunk or limbs, alveolar subtype, and the presence of PAX-FOXO1 carry a worse prognosis. Other prognostic factors include TNM stage, local invasion, parameningeal site, and lack of response to induction therapies [58,59,60].

The first histology-specific nomogram for RMS was developed in 2011 by Chisholm et al. It estimates the probability of curing patients with nonmetastatic RMS who relapsed after achieving complete local control with initial therapy [59]. This type of computation might help physicians to distinguish patients with a reasonable chance of being cured with salvage therapy from those with a poor chance of salvage that should better be directed toward experimental or palliative therapy. Of note, the authors could not assess the role of resectability as a prognostic factor at the time of relapse, although in this patient, the chance to deliver local therapy remains critical.

In 2014, Yang et al. developed and internally validated a nomogram to predict 5- and 10-year overall survival and median survival time in children and adolescents with RMS using population-based data collected by the Surveillance, Epidemiology, and End Results (SEER) program of the National Cancer Institute. The nomogram relies on easily available tumor and patient-related variables as well as treatment variables, i.e., surgical treatment and RT delivery, and was built on a large series of 1679 patients. Nevertheless, it has some important limitations related to the SEER database. In fact, this public dataset does not include information on ChT delivery, surgical margins, or patients’ comorbidities, all of which are regarded as important prognostic factors in this disease. Furthermore, the SEER program does not provide a central pathology review. In addition, the cohort covers a wide time span (1990–2010) during which improvements in the management of these tumors possibly improved the prognosis of these patients [61].

Two further nomograms were built using the SEER database (Table 3)—and are therefore burdened by the same limitations—to predict 5- and 10-year cause-specific survival and median survival time in patients who underwent surgery alone and surgery + adjuvant RT [61]. The nomograms include histological subtype and the presence of positive regional lymph nodes, and they underwent good internal validation.

#### 3.4.4. Desmoid-Type Fibromatosis

Desmoid-type fibromatosis is a rare mesenchymal neoplasm with no metastatic potential but unpredictable local behavior, potentially occurring at any site. Typically, patients with desmoid-type fibromatosis present with a mass that has become symptomatic due to local compression symptoms that vary depending on the site of occurrence: pain due to nerve compression or invasion, digestive symptoms in abdominal desmoid-type fibromatosis, functional morbidity in desmoid-type fibromatosis of the extremity. In 5–15% of cases, desmoid-type fibromatosis arises in patients with familiar adenomatous polyposis (APC gene mutation), while the rest of cases are sporadic and characterized by a mutation in the CTNNB1 gene. Both mutations result in abnormal β-catenin accumulation within the cell.

Surgery was long considered the standard of care for the initial treatment of resectable disease. However, the unpredictable biological behavior of desmoid tumors, with relatively high rates of spontaneous regression or stabilization, the very high rates of recurrence post-resection, and the significant morbidity of surgery, prompted the international community to re-evaluate non-surgical strategies for their management. In fact, in light of the recent evidence, the ESMO and Desmoid Tumor Working Group (DTWG) guidelines support a wait-and-see frontline approach to observe tumor behavior over time [62,63]. Medical treatments should be proposed in cases of symptomatic or progressive disease, with several therapeutic options available (kinase inhibitors and cytotoxic agents); while surgery is now recommended only in cases of complications or failure of medical treatments.

In 2013, Crago et al. from the MSKCC developed and externally validated a nomogram to predict postoperative 3-year, 5-year, and 7-year LR-free survival [64]. Interestingly, the computation is built on variables that are all available preoperatively (Table 3), making the nomogram a valid tool to support management setup from the first visit. Some authors found an association between surgical margin status, β-catenin mutation, and disease recurrence [65,66]. In the MSKCC nomogram, the β-catenin mutation was not evaluated, while margin status was not significantly associated with LRFS at multivariable analysis. Only in a sub-group analysis of patients with small tumors (<5 cm), R1 resection was found to be significantly associated with disease recurrence. However, in this subset of patients, recurrence was rare, supporting a watchful-waiting strategy more than an aggressive approach to achieve complete microscopic resection in small tumors that received R1 surgery.

With this nomogram, patients with a high risk of local recurrence might be easily selected to receive other therapeutic options in the case of disease progression or symptomatic disease. However, the shift towards a non-surgical and wait-and-see approach calls for the development of a nomogram able to predict the chance of disease progression in non-operated patients.

**Table 3:** Histology-Specific and Histology-Specific and Site-Specific Nomograms for Patients With STS.

	Study	Development Series Characteristics			Nomogram Details			Internal Validation	External Validation	
		Selection Criteria	Timeframe	No. of Centers	Predicted Outcomes	No. of Patients	Nomogram’s Covariates (a)	Concordance Index	Yes/No	Concordance Index
Liposarcoma	Dalal [55] 2006	Nonmetastatic liposarcoma of the extremity, trunk, or retroperitoneum	1982–2005	1	5-y and 12-y DSS	801	Histology (5 categories), tumor burden (continuous), age (continuous), surgical resection margins (R0 vs. R1 vs. R2), site (5 categories), presentation status (prior excision vs. biopsy vs. no treatment), tumor depth (superficial vs. deep), sex (male vs. female)	0.83	No	
Synovial sarcoma	Canter [57] 2008	Primary, localized, surgically treated patients with synovial sarcoma who did not receive AI chemotherapy	1982–2006	1	3-y and 5-y DSS	196	Size (continuous), site (upper extremity vs. lower extremity vs. others), depth (superficial vs. deep), variant (biphasic vs. monophasic)	0.77	No	-
Rhabdomyo-sarcoma	Yang [60] 2014	Patients with primary RMS (both localized or metastatic) and aged birth to 19 y	1990–2010	SEER database (populationbaseddata set)	5-y and 10-y OS, median survival time	1679	Tumor stage (localized vs. regional vs. distant), surgery (yes vs. no), RT (yes vs. no), size (continuous), histological subtype (alveolar vs. embryonal vs. others), age (continuous), tumor site (favorable vs. unfavorable)	0.74	No	
	Shen [61] 2014	Patients with primary RMS treated with surgery (all ages)	1990–2010	SEER database (populationbaseddata set)	5-y and 10-y causespecific survival and median survival time for patients treated with surgery alone or with surgery plus RT	1578	Age (continuous), size (continuous), stage (localized vs. regional vs. distant), histological subtype (embryonal vs. alveolar vs. pleomorphic vs. others), positive regional lymph nodes (no lymph nodes examined vs. 0 vs. 1–3 vs. 4)	0.78	No	
	Chisholm [59] 2011	Children with nonmetastatic RMS and embryonal RMS who developed disease recurrence after achieving complete local control (complete remission or stable mass for >6 mo after the end of therapy) with primary therapy	1984–2003 (primary treatment)	Multicentric (international registry)	Probability of cure defined as survival 3.0 y after disease recurrence	474	Type of recurrence (local vs. metastatic 6 local), prior RT (yes vs. no), type of chemotherapy (2-drug vs. 3-drug vs. 6-drug), lymph node status (N0 vs. N1 vs. Nx), tumor size (missing vs. <5 cm vs. >5 cm), tumor site (favorable vs. unfavorable), histology (alveolar vs. nonalveolar), time to disease recurrence (>1.5 y vs. <1.5 y)		No	
Desmoid-type fibromatosis	Crago [64] 2013	Surgically treated desmoid-type fibromatosis	1982–2011	1	3-y, 5-y, and 7-y LRFS and median time to LR	495	Age (continuous), tumor site (extremity vs. chest wall vs. GI/intrabdominal vs. other vs. abdominal wall), size (continuous)	0.70	Yes	0.66 (b)
Breast phyllodes tumors	Tan [67] 2012	Surgically treated phyllodes tumors of the breast	1992–2010	1	1-y, 3-y, 5-y, and 10-y RFS	552	Surgical resection margin (negative vs. positive), mitosis per 10 high-power fields (continuous), atypia (marked vs. moderate vs. mild), overgrowth (present vs. absent)	0.79	No	
Uterine leiomyo-sarcoma	Zivanovic [68] 2012	Surgically treated uterine leiomyosarcoma	1982–2008	1	5-y OS	185	Mitotic index (continuous), tumor grade (high vs. not high), locoregional metastasis (yes vs. no), distant metastasis (yes vs. no), tumor size (continuous), cervical involvement (yes vs. no), age at diagnosis (continuous)	0.67	Yes	0.67 (c)

Abbreviations: AI, anthracycline-ifosfamide; DSS, disease-specific survival; GI, gastrointestinal; LR, local recurrence; LRFS, local recurrence-free survival; OS, overall survival; RFS, recurrence-free survival; RMS, rhabdomyosarcoma; RT, radiotherapy; SEER, Surveillance, Epidemiology, and End Results; STS, soft tissue sarcoma. (a) The variables are listed according to their nomogram score range, which reflects their relative effect on the predicted outcome, on a decreasing basis (the first variable exerts the strongest influence on the predicted outcome). (b) External validation was performed on 274 patients with desmoid-type fibromatosis who underwent complete surgical resection in 24 cancer centers from the French Sarcoma Group (Harrell C-index, 0.659, [95% confidence interval, 0.598–0.712] [54]. (c) External validation was performed on 187 patients treated with hysterectomy between 1994 and 2010 at Brigham and Women’s Hospital/Dana-Farber Cancer Institute in Boston, Massachusetts, and the European Institute of Oncology in Milan, Italy (Harrell C-index, 0.67 [95% confidence interval, 0.62–0.72]) [57].

### 3.5. Histology-Specific and Site-Specific Nomograms

#### 3.5.1. Uterine Leiomyosarcoma

Uterine leiomyosarcoma (ULMS) is the most frequent type of uterine sarcoma and accounts for about 5% of all uterine cancers. Though rare, the disease carries a poor prognosis with high rates of local and distant recurrence. Surgical resection (i.e., hysterectomy with complete surgical resection of all gross tumors) is the mainstay of treatment in all resectable cases. Radiotherapy and/or chemotherapy can be considered according to the stage at time of diagnosis. However, no adjuvant treatment strategy has demonstrated a survival benefit, and high rates of recurrence and progression are registered despite standard therapies.

Traditionally, ULMS are classified using the Federation Internationale de Gynecologie et d’Obstetrique (FIGO) staging system developed in 2009 or the AJCC/UICC staging system for STS. The FIGO staging system identifies four stages according to tumor size, extension with respect to the uterus and pelvic organs, and the presence/absence of DM, while the AJCC staging system is based on tumor dimension, regional lymph node involvement, and the presence of DM.

In 2012, Zivanovic et al. [68] from the MSKCC developed and internally validated a novel ULMS-specific nomogram that combines seven variables (age, grade, tumor size, mitotic rate, presence of cervical invasion, presence of locoregional metastases, and presence of distant metastases) to predict post-resection 5-year overall survival (OS). This nomogram was demonstrated to be more accurate in predicting overall survival compared to the FIGO and AJCC staging systems.

Of note, it cannot be applied to patients not eligible for surgery (due to advanced disease or poor general performance status) because the development cohort included only surgically treated patients. The nomogram underwent external validation on a series from the Brigham and Women’s Hospital/Dana-Farber Cancer Institute and the European Institute of Oncology, demonstrating good discrimination (Harrell C-index, 0.67) and calibration [69].

#### 3.5.2. Breast Phyllodes Tumors

Breast phyllodes tumors (BFT) are a rare group of mammary fibroepithelial tumors with recurrent and metastatic potential and a wide spectrum of morphologies. The WHO classification stratifies BFT into three categories (benign, borderline, and malignant) on the basis of several histologic features (stromal features of cytologic atypia, mitotic activity, degree of hypercellularity, overgrowth, and nature of tumor margins, and the presence of heterologous stromal elements) [70]. Of note, benign BFTs share some histological features with innocuous cellular fibroadenoma, with which they can therefore be erroneously mistaken. Furthermore, malignant BFT resembles and may be misdiagnosed with primary breast sarcoma or spindle cell metaplastic carcinoma. Although the correlation between histological features and the outcome is clear, the identification of an accurate and reproducible grading system is challenging because each of these parameters might be weighted differently by different pathologists. In addition, there is no consensus about the prognostic weight of each histological feature and how they should be combined.

In 2012, a nomogram based on degree of stromal atypia, stromal mitoses per 10 high-power fields, stromal overgrowth, and surgical resection margins (AMOS criteria) was developed by Tan et al. [67] from Singapore General Hospital to predict recurrence-free survival at 1, 3, 5, and 10 years. With this nomogram, authors have partially managed to overcome the limits of the WHO classification.

## 4. Future Perspectives

With the aim of improving the accuracy and discriminative ability of available tools for prognosis prediction, recent research focused on the identification of new prognostic markers of outcome, and particular attention was recently put on genomic, radiomic, or immunologic markers.

Although FNCLCC grading is one of the best available predictors of oncological outcomes and, as such, is included in several nomograms, it is still limited by its reproducibility from one pathologist to another and by the fact that it forces each tumor into one of three categories.

In contrast, the identification of genomic markers of outcome (i.e., specific genes or DNA sequences associated with outcome) might potentially give a more granular and accurate picture of the different behaviors of sarcomas, and their integration into outcome prediction tools might improve their prognostic power.

In 2010, Chibon et al. identified a gene expression signature composed of 67 genes associated with mitosis and chromosome management—named Complexity Index in SARComas (CINSARC)—able to predict metastatic outcome in non-translocation-related sarcomas [71]. CINSARC was also successfully validated in a second independent cohort by the same group [72]. In this retrospective series, CINSARC had a better discriminative power compared to FNCLCC grading; in particular, CINSARC split into two groups with different metastatic potential tumors considered grade 2 according to FNCLCC. These results were partially refuted by Frezza et al., who did not observe any relevant correlation between CINSARC groups and outcome in an independent series of high-risk patients with localized STS treated with preoperative chemotherapy within a prospective, randomized, phase III study (ISG-STS 1001 study) [73]. Indeed, a formal, prospective independent validation of this signature is still missing. In addition, to what extent the addition of CINSARC to Sarculator improves its prognostication ability is left to be understood.

Of note, the French Sarcoma Group is running a prospective, randomized, phase 3 trial to explore the potential benefit of chemotherapy in high-risk CINSARC patients and to prospectively validate the prognostic role of CINSARC in FNCLCC grade 1 and 2 STS [72]. However, while CINSARC may have a prognostic value, no information is presently available regarding its capability to predict the effect of anthracycline plus ifosfamide chemotherapy, while Sarculator did show to be able to do so.

In the last decade, growing attention has been put on the potential role of radiomic analysis. Radiomics consists in the extraction of a large number of quantitative data from radiologic imaging by the application of sophisticated software with artificial intelligence. These data are intended to reveal tumoral patterns and characteristics that are not visible to the naked eye and may significantly contribute to the diagnosis, management, and prognosis of tumors. The identification of radiomic signatures able to predict prognosis is in its early phases, and while the first studies have been recently reported, none of them has been validated and incorporated into clinical practice. Of note, one of the most important limitations of radiomics is its reproducibility when it comes to the way the exam was performed, the IV contrast medium delivered, etc.

Similar to radiology, a shift from traditional pathology toward the use of artificial intelligence-based systems for the analysis of tissue samples is becoming an increasingly likely prospect. With the application of machine learning techniques, digital pathology with whole-slide imaging enables the capture of information far beyond that obtained with traditional pathology. However, although it represents a promising tool for the diagnosis and clinical management of sarcoma patients, its application is just at the very beginning and needs to be further explored in the future [74,75].

Immunologic markers are also being studied as prognosticators in sarcoma, but we are far from having developed a similar tool, such as the inflammosome in colonic cancer [76], for sarcoma.

Finally, the impact of multimodal therapy on outcome is not incorporated in any of the available nomograms, as it has been performed, for example, in breast cancer.

This requires data from larger patient data sets than the ones used so far to build the available tools. A large series of patients with sarcoma requires large collaborations. Large collaborations, networks, working groups, and registries have all been critical for advancing knowledge in recent years and will continue to be so, even as access to real-world data makes it easier to collect information and run prognosis and outcome studies.

The authors should discuss the results and how they can be interpreted from the perspective of previous studies and the working hypotheses. The findings and their implications should be discussed in the broadest possible context. Future research directions may also be highlighted.

## Figures and Tables

**Table 2:** Nomograms for Patients With RPS.

Study	Development Series Characteristics			Nomogram Details			Internal Validation	External Validation	
	Selection Criteria	Timeframe	No. of Centers	Predicted Outcomes	No. of Patients	Nomogram’s Covariates (a)	Concordance Index	Yes/No	Concordance Index
Anaya [48] 2010	Primary or recurrent, nonmetastatic, resected	1996–2006	1	Median OS, 3-y OS, and 5-y OS	343	Histology (3 categories), completeness of surgical resection, age (dichotomic; cutoff at 65 y), multifocality, tumor size (dichotomic; cutoff at 15 cm), presentation (primary vs. recurrent)	0.73 (95% CI, 0.71–0.75)	No	-
Ardoino [10] 2010	Primary, localized, resected	1985–2007	1	5-y OS and 10-y OS	192	Histology (5 categories), FNCLCC grade, size (continuous), surgical resection margins (complete vs. incomplete), age (continuous)	0.73	No	-
Gronchi [2] 2013	Primary, localized, resected	1999–2009	3	7-y OS	523	FNCLCC grade, tumor size (continuous), histology (7 categories), patient age (continuous), multifocality (yes vs. no), extent of surgical resection (complete vs. incomplete)	0.74	Yes	0.67–0.73 (b)
				7-y DFS	475	FNCLCC grade, tumor size (continuous), histology (7 categories), multifocality (yes vs. no)	0.71	Yes	0.63–0.69 (b)
Tan [24] 2016	Primary, localized, resected	1982–2010	1	3-y, 5-y, and 10-y DSD	632	Histology (7 categories), extent of surgical resection (R0/R1 vs. R2), no. of organs resected (dichotomic, cutoff at 3 organs), size (3 categories), RT (yes vs. no)	0.71 (95% CI, 0.66–0.74)	No	-
				3-y, 5-y, and 10-y LR rate	574	Histology (7 categories), size (3 categories), age (dichotomic; cutoff at 65 y), surgical resection (R0 vs. R1), location (pelvis vs. other), vascular resection (yes vs. no), no. of resected organs (dichotomic; cutoff at 3 organs)	0.71 (95% CI, 0.67–0.75)	No	-
				3-y, 5-y, and 10-y DR rate	632	Histology (7 categories), no. of resected organs (0 vs. 1–2 vs. 3 organs), size (3 categories), RT (yes vs. no), vascular resection (yes vs. no)	0.74 (95% CI, 0.69–0.77)	No	-
Raut [49] 2019	First local relapse, nonmetastatic, resected with curative intent	2002–2011	22 (TARPSWG centers)	6-y DFS	602	Histology (6 categoies), number of resected organs at first surgery (0–7), multifocality at second surgery (yes vs. no), quality of surgery at second surgery (complete vs. incomplete), grade (1/2 vs. 3), ChT after first surgery (yes vs. no), RT after first surgery (yes vs. no)	0.67	No	-
			22 (TARPSWG centers)	6-y OS	602	Histology (6 categories), age at second surgery (continuous), quality of surgery at second surgery (complete vs. incomplete), number of resected organs at first surgery (0–7), grade (1/2 vs. 3), multifocality at second surgery (yes vs. no), RT after first surgery (yes vs. no)	0.70	No	-
Callegaro [13] 2021	Primary (non-recurrent), non-metastatic, resected with curative intent	2002–2017	4	5-y OS at different time points (0–60 months) throughout the first 5 years of follow-up	1357	Landmark time, FNCLCC grade (1/2/3), occurrence of LR (yes vs. no), occurrence of DM (yes vs. no), age at first surgery (continuous), and completeness of resection (complete vs. incomplete)	0.75–0.85	Yes	0.72–0.79 (c)
			4	5-y DFS at different time points (0–60 months) throughout the first 5 years of follow-up	1357	Tumour size (continuous), FNCLCCgrade (1/2/3), multifocality (yes vs. no), landmark time, histology and the interaction terms between landmark time and tumour size, tumour grade, and multifocality.	0.64–0.72	Yes	0.62–0.68 (c)

Abbreviations: 95% CI, 95% confidence interval; ChT, chemotherapy; DFS, disease-free survival; DR, distant recurrence; DSD, disease-specific death; FNCLCC, Federation Francaise des Centres de Lutte Contre le Cancer; LR, local; recurrence; OS, overall survival; RPS, retroperitoneal sarcoma; RT, radiotherapy; TARPSWG, TransAtlantic Retroperitoneal Sarcoma Working Group. (a) Variables are listed according to the size of their score range, which reflects their relative effect on the predicted outcome, on a decreasing basis (the first variable exerts the strongest influence on the predicted outcome). (b) External validations were performed on 135 patients from the Institut Gustave Roussy in Villejuif, France (Harrell C-index of 0.67 for the OS nomogram and 0.68 for the DFS nomogram) [2]; 631 patients from a multicentric cohort of the Trans-Atlantic Retroperitoneal Sarcoma Working Group (Harrell C-index of 0.73 for the OS nomogram and 0.69 for the DFS nomogram) [50]; 144 patients from Taipei Veterans General Hospital in Taipei, Taiwan (Harrell C-index, 0.72 for the OS nomogram) [51]; 502 patients who underwent resection of primary RPS at nine high-volume academic US institutions (data from the US Sarcoma Collaborative database) (Harrell C-index of 0.69 for the OS nomogram and 0.65 for the DFS nomogram) [25]; and 109 patients who underwent complete resection for primary retroperitoneal sarcoma at the National Cancer Centre Singapore (Harrell C-index of 0.73 fot the OS nomogram and 0.63 for the DFS nomogram.) [26]. (c) External validation was performed on 487 patients for OS and 452 patients for DFS aged ≥16 years affected by primary (non-recurrent), non-metastatic, RPS who underwent surgery with curative intent at Brigham and Women’s Hospital, Dana-Farber Cancer Institute, Harvard Medical School (Boston, MA, USA), Institut Bergonie (Bordeaux, France), Institut Curie (Paris, France), Leiden University Medical Center (Leiden, The Netherlands), Maria Sklodowska-Curie Institute-Oncology Center (Warsaw, Poland), and The Netherlands Cancer Institute (Amsterdam, The Netherlands).

## References

[1] Kattan M.W., Hess K.R., Amin M.B., Lu Y., Moons K.G., Gershenwald J.E., Gimotty P.A., Guinney J.H., Halabi S., Lazar A.J. (2016). members of the AJCC Precision Medicine Core. American Joint Committee on Cancer acceptance criteria for inclusion of risk models for individualized prognosis in the practice of precision medicine. CA Cancer J. Clin..

[2] Gronchi A., Miceli R., Shurell E., Eilber F.C., Eilber F.R., Anaya D.A., Kattan M.W., Honoré C., Lev D.C., Colombo C. (2013). Outcome prediction in primary resected retroperitoneal soft tissue sarcoma: Histology-specific overall survival and disease-free survival nomograms built on major sarcoma center data sets. J. Clin. Oncol..

[3] Edge S.B., Byrd D., Compton C.C., Fritz A.G., Greene F.L., Trotti A. (2010). AJCC Cancer Staging Manual.

[4] Yoon S.S. (2018). The New American Joint Commission on Cancer Staging System for Soft Tissue Sarcomas: Splitting versus Lumping. Ann. Surg. Oncol..

[5] Maki R.G., Moraco N., Antonescu C.R., Hameed M., Pinkhasik A., Singer S., Brennan M.F. (2013). Toward better soft tissue sarcoma staging: Building on american joint committee on cancer staging systems versions 6 and 7. Ann. Surg. Oncol..

[6] Lahat G., Tuvin D., Wei C., Anaya D.A., Bekele B.N., Lazar A.J., Pisters P.W., Lev D., Pollock R.E. (2008). New perspectives for staging and prognosis in soft tissue sarcoma. Ann. Surg. Oncol..

[7] Cates J.M.M. (2017). Performance Analysis of the American Joint Committee on Cancer 8th Edition Staging System for Retroperitoneal Sarcoma and Development of a New Staging Algorithm for Sarcoma-Specific Survival. Ann. Surg. Oncol..

[8] Fisher S.B., Chiang Y.J., Feig B.W., Cormier J.N., Hunt K.K., Torres K.E., Roland C.L. (2019). An Evaluation of the Eighth Edition of the American Joint Committee on Cancer (AJCC) Staging System for Retroperitoneal Sarcomas Using the National Cancer Data Base (NCDB): Does Size Matter?. Am. J. Clin. Oncol..

[9] Cates J.M.M. (2018). The AJCC 8th Edition Staging System for Soft Tissue Sarcoma of the Extremities or Trunk: A Cohort Study of the SEER Database. J. Natl. Compr. Cancer Netw..

[10] Pasquali S., Palmerini E., Quagliuolo V., Martin-Broto J., Lopez-Pousa A., Grignani G., Brunello A., Blay J.Y., Tendero O., Diaz-Beveridge R. (2022). Neoadjuvant chemotherapy in high-risk soft tissue sarcomas: A Sarculator-based risk stratification analysis of the ISG-STS 1001 randomized trial. Cancer.

[11] Gronchi A., Palmerini E., Quagliuolo V., Martin Broto J., Lopez Pousa A., Grignani G., Brunello A., Blay J.Y., Tendero O., Diaz Beveridge R. (2020). Neoadjuvant Chemotherapy in High-Risk Soft Tissue Sarcomas: Final Results of a Randomized Trial From Italian (ISG), Spanish (GEIS), French (FSG), and Polish (PSG) Sarcoma Groups. J. Clin. Oncol..

[12] Callegaro D., Miceli R., Bonvalot S., Ferguson P.C., Strauss D.C., van Praag V.V.M., Levy A., Griffin A.M., Hayes A.J., Stacchiotti S. (2019). Development and external validation of a dynamic prognostic nomogram for primary extremity soft tissue sarcoma survivors. Eclinicalmedicine.

[13] Callegaro D., Barretta F., Swallow C.J., Strauss D.C., Bonvalot S., Honorè C., Stoeckle E., van Coevorden F., Haas R., Rutkowski P. (2021). Longitudinal prognostication in retroperitoneal sarcoma survivors: Development and external validation of two dynamic nomograms. Eur. J. Cancer.

[14] Rueten-Budde A.J., van Praag V.M., van de Sande M.A.J., Fiocco M., PERSARC Study Group (2021). External validation and adaptation of a dynamic prediction model for patients with high-grade extremity soft tissue sarcoma. J. Surg. Oncol..

[15] Kattan M.W., Leung D.H., Brennan M.F. (2002). Postoperative nomogram for 12-year sarcoma-specific death. J. Clin. Oncol..

[16] Mariani L., Miceli R., Kattan M.W., Brennan M.F., Colecchia M., Fiore M., Casali P.G., Gronchi A. (2005). Validation and adaptation of a nomogram for predicting the survival of patients with extremity soft tissue sarcoma using a three-grade system. Cancer.

[17] Eilber F.C., Brennan M.F., Eilber F.R., Dry S.M., Singer S., Kattan M.W. (2004). Validation of the postoperative nomogram for 12-year sarcoma-specific mortality. Cancer.

[18] Eilber F.C., Kattan M.W. (2007). Sarcoma nomogram: Validation and a model to evaluate impact of therapy. J. Am. Coll. Surg..

[19] Szkandera J., Gerger A., Liegl-Atzwanger B., Absenger G., Stotz M., Samonigg H., Maurer-Ertl W., Stojakovic T., Ploner F., Leithner A. (2013). Validation of the prognostic relevance of plasma C-reactive protein levels in soft-tissue sarcoma patients. Br. J. Cancer.

[20] Szkandera J., Pichler M., Liegl-Atzwanger B., Absenger G., Stotz M., Ploner F., Stojakovic T., Samonigg H., Eberhard K., Leithner A. (2014). The elevated pre-operative plasma fibrinogen level is an independent negative prognostic factor for cancer-specific, disease-free and overall survival in soft-tissue sarcoma patients. J. Surg. Oncol..

[21] Ng D.W.J., Tan G.H.C., Chia C.S., Lim C.X., Chee S.K., Quek R.H.H., Farid M., Teo M.C.C. (2017). Is the Memorial Sloan Kettering Cancer Centre (MSKCC) sarcoma nomogram useful in an Asian population?. Asia-Pac. J. Clin. Oncol..

[22] Bagaria S.P., Wagie A.E., Gray R.J., Pockaj B.A., Attia S., Habermann E.B., Wasif N. (2015). Validation of a Soft Tissue Sarcoma Nomogram Using a National Cancer Registry. Ann. Surg. Oncol..

[23] Shuman A.G., Brennan M.F., Palmer F.L., Kuk D., Moraco N., Singer S., Shah J.P., Patel S.G. (2015). Soft tissue sarcoma of the head & neck: Nomogram validation and analysis of staging systems. J. Surg. Oncol..

[24] Tan M.C., Brennan M.F., Kuk D., Agaram N.P., Antonescu C.R., Qin L.X., Moraco N., Crago A.M., Singer S. (2016). Histology-based Classification Predicts Pattern of Recurrence and Improves Risk Stratification in Primary Retroperitoneal Sarcoma. Ann. Surg..

[25] Squires M.H., Ethun C.G., Donahue E.E., Benbow J.H., Anderson C.J., Jagosky M.H., Salo J.C., Hill J.S., Ahrens W., Prabhu R.S. (2021). A multi-institutional validation study of prognostic nomograms for retroperitoneal sarcoma. J. Surg. Oncol..

[26] Wong R.X., Koh Y.S., Ong F., Farid M., Tay T.K.Y., Teo M. (2020). Applicability of the Sarculator and MSKCC nomograms to retroperitoneal sarcoma prognostication in an Asian tertiary center. Asian J. Surg..

[27] Ferrari A., Miceli R., Casanova M., Gronchi A., Collini P., Meazza C., Zaffignani E., Massimino M., Spreafico F., Mariani L. (2007). Adult-type soft tissue sarcomas in paediatric age: A nomogram-based prognostic comparison with adult sarcoma. Eur. J. Cancer.

[28] Szkandera J., Gerger A., Liegl-Atzwanger B., Absenger G., Stotz M., Friesenbichler J., Trajanoski S., Stojakovic T., Eberhard K., Leithner A. (2013). The lymphocyte/monocyte ratio predicts poor clinical outcome and improves the predictive accuracy in patients with soft tissue sarcomas. Int. J. Cancer.

[29] Kandel R.A., Bell R.S., Wunder J.S., O′Sullivan B., Catton C.N., White L.M., Davis A.M. (1999). Comparison between a 2- and 3-grade system in predicting metastatic-free survival in extremity soft-tissue sarcoma. J. Surg. Oncol..

[30] Danieli M., Barretta F., Fiore M., Radaelli S., Sangalli C., Barisella M., Stacchiotti S., Palassini E., Miceli R., Frezza A.M. (2022). Refining the Approach to Patients with Primary Soft Tissue Sarcoma of the Extremities and Trunk Wall: Outcome Improvement Over Time at a Single Institution. Ann. Surg. Oncol..

[31] Squires M.H., Ethun C.G., Donahue E.E., Benbow J.H., Anderson C.J., Jagosky M.H., Manandhar M., Patt J.C., Kneisl J.S., Salo J.C. (2022). Extremity Soft Tissue Sarcoma: A Multi-Institutional Validation of Prognostic Nomograms. Ann. Surg. Oncol..

[32] Cahlon O., Brennan M.F., Jia X., Qin L.X., Singer S., Alektiar K.M. (2012). A postoperative nomogram for local recurrence risk in extremity soft tissue sarcomas after limb-sparing surgery without adjuvant radiation. Ann. Surg..

[33] Bonvalot S., Levy A., Terrier P., Tzanis D., Bellefqih S., Le Cesne A., Le Péchoux C. (2017). Primary Extremity Soft Tissue Sarcomas: Does Local Control Impact Survival?. Ann. Surg. Oncol..

[34] Callegaro D., Miceli R., Bonvalot S., Ferguson P., Strauss D.C., Levy A., Griffin A., Hayes A.J., Stacchiotti S., Pechoux C.L. (2016). Development and external validation of two nomograms to predict overall survival and occurrence of distant metastases in adults after surgical resection of localised soft-tissue sarcomas of the extremities: A retrospective analysis. Lancet Oncol..

[35] Digital Forest srl. http://play.google.com/store/apps/details?id=it.digitalforest.sarculator.

[36] Voss R.K., Callegaro D., Chiang Y.J., Fiore M., Miceli R., Keung E.Z., Feig B.W., Torres K.E., Scally C.P., Hunt K.K. (2022). Sarculator is a Good Model to Predict Survival in Resected Extremity and Trunk Sarcomas in US Patients. Ann. Surg. Oncol..

[37] Pasquali S., Pizzamiglio S., Touati N., Litiere S., Marreaud S., Kasper B., Gelderblom H., Stacchiotti S., Judson I., Dei Tos A.P. (2019). The impact of chemotherapy on survival of patients with extremity and trunk wall soft tissue sarcoma: Revisiting the results of the EORTC-STBSG 62931 randomised trial. Eur. J. Cancer.

[38] van Praag V.M., Rueten-Budde A.J., Jeys L.M., Laitinen M.K., Pollock R., Aston W., van der Hage J.A., Dijkstra P.D.S., Ferguson P.C., Griffin A.M. (2017). A prediction model for treatment decisions in high-grade extremity soft-tissue sarcomas: Personalised sarcoma care (PERSARC). Eur. J. Cancer.

[39] Rueten-Budde A.J., van Praag V.M., van de Sande M.A.J., Fiocco M., PERSARC study group (2018). Dynamic prediction of overall survival for patients with high-grade extremity soft tissue sarcoma. Surg. Oncol..

[40] Gronchi A., Strauss D.C., Miceli R., Bonvalot S., Swallow C.J., Hohenberger P., Van Coevorden F., Rutkowski P., Callegaro D., Hayes A.J. (2016). Variability in Patterns of Recurrence After Resection of Primary Retroperitoneal Sarcoma (RPS): A Report on 1007 Patients From the Multi-institutional Collaborative RPS Working Group. Ann. Surg..

[41] Gronchi A., Miceli R., Allard M.A., Callegaro D., Le Péchoux C., Fiore M., Honoré C., Sanfilippo R., Coppola S., Stacchiotti S. (2015). Personalizing the approach to retroperitoneal soft tissue sarcoma: Histology-specific patterns of failure and postrelapse outcome after primary extended resection. Ann. Surg. Oncol..

[42] Toulmonde M., Bonvalot S., Ray-Coquard I., Stoeckle E., Riou O., Isambert N., Bompas E., Penel N., Delcambre-Lair C., Saada E. (2014). French Sarcoma Group. Retroperitoneal sarcomas: Patterns of care in advanced stages, prognostic factors and focus on main histological subtypes: A multicenter analysis of the French Sarcoma Group. Ann. Oncol..

[43] Singer S., Antonescu C.R., Riedel E., Brennan M.F., Pollock R.E. (2003). Histologic Subtype and Margin of Resection Predict Pattern of Recurrence and Survival for Retroperitoneal Liposarcoma. Ann. Surg..

[44] Lahat G., Anaya D.A., Wang X., Tuvin D., Lev D., Pollock R.E. (2008). Resectable well-differentiated versus dedifferentiated liposarcomas: Two different diseases possibly requiring different treatment approaches. Ann. Surg. Oncol..

[45] Pasquali S., Gronchi A., Strauss D., Bonvalot S., Jeys L., Stacchiotti S., Hayes A., Honore C., Collini P., Renne S.L. (2016). Resectable extra-pleural and extra-meningeal solitary fibrous tumours: A multi-centre prognostic study. Eur. J. Surg. Oncol..

[46] Gronchi A., Collini P., Miceli R., Valeri B., Renne S.L., Dagrada G., Fiore M., Sanfilippo R., Barisella M., Colombo C. (2015). Myogenic differentiation and histologic grading are major prognostic determinants in retroperitoneal liposarcoma. Am. J. Surg. Pathol..

[47] Callegaro D., Raut C.P., Ng D., Strauss D.C., Honoré C., Stoeckle E., Bonvalot S., Haas R.L., Vassos N., Conti L. (2021). Has the Outcome for Patients Who Undergo Resection of Primary Retroperitoneal Sarcoma Changed Over Time? A Study of Time Trends During the Past 15 years. Ann. Surg. Oncol..

[48] Anaya D.A., Lahat G., Wang X., Xiao L., Pisters P.W., Cormier J.N., Hunt K.K., Feig B.W., Lev D.C., Pollock R.E. (2010). Postoperative nomogram for survival of patients with retroperitoneal sarcoma treated with curative intent. Ann. Oncol..

[49] Raut C.P., Callegaro D., Miceli R., Barretta F., Rutkowski P., Blay J.Y., Lahat G., Strauss D.C., Gonzalez R., Ahuja N. (2019). Predicting Survival in Patients Undergoing Resection for Locally Recurrent Retroperitoneal Sarcoma: A Study and Novel Nomogram from TARPSWG. Clin. Cancer Res..

[50] Raut C.P., Miceli R., Strauss D.C., Swallow C.J., Hohenberger P., van Coevorden F., Rutkowski P., Fiore M., Callegaro D., Casali P.G. (2016). External validation of a multi-institutional retroperitoneal sarcoma nomogram. Cancer.

[51] Chou Y.S., Liu C.Y., Chang Y.H., King K.L., Chen P.C., Pan C.C., Shen S.H., Liu Y.M., Lin A.T., Chen K.K. (2016). Prognostic factors of primary resected retroperitoneal soft tissue sarcoma: Analysis from a single asian tertiary center and external validation of gronchi’s nomogram. J. Surg. Oncol..

[52] Nessim C., Raut C.P., Callegaro D., Barretta F., Miceli R., Fairweather M., Rutkowski P., Blay J.Y., Strauss D., Gonzalez R. (2021). Postoperative Morbidity After Resection of Recurrent Retroperitoneal Sarcoma: A Report from the Transatlantic Australasian RPS Working Group (TARPSWG). Ann. Surg. Oncol..

[53] Seidensaal K., Kieser M., Hommertgen A., Jaekel C., Harrabi S.B., Herfarth K., Mechtesheimer G., Lehner B., Schneider M., Nienhueser H. (2021). Neoadjuvant irradiation of retroperitoneal soft tissue sarcoma with ions (Retro-Ion): Study protocol for a randomized phase II pilot trial. Trials.

[54] Ng D., Cyr D.P., Burtenshaw S.M., Callegaro D., Gronchi A., Shultz D., Brar S., Chung P., Gladdy R.A., Catton C. (2022). Effect of Preoperative Treatment on the Performance of Predictive Nomograms in Primary Retroperitoneal Sarcoma. Ann. Surg. Oncol..

[55] Dalal K.M., Kattan M.W., Antonescu C.R., Brennan M.F., Singer S. (2006). Subtype specific prognostic nomogram for patients with primary liposarcoma of the retroperitoneum, extremity, or trunk. Ann. Surg..

[56] Stacchiotti S., Van Tine B.A. (2018). Synovial Sarcoma: Current Concepts and Future Perspectives. J. Clin. Oncol..

[57] Canter R.J., Qin L.X., Maki R.G., Brennan M.F., Ladanyi M., Singer S. (2008). A synovial sarcoma-specific preoperative nomogram supports a survival benefit to ifosfamide-based chemotherapy and improves risk stratification for patients. Clin. Cancer Res..

[58] Gartrell J., Pappo A. (2020). Recent advances in understanding and managing pediatric rhabdomyosarcoma. F1000Research.

[59] Chisholm J.C., Marandet J., Rey A., Scopinaro M., de Toledo J.S., Merks J.H., O′Meara A., Stevens M.C., Oberlin O. (2011). Prognostic factors after relapse in nonmetastatic rhabdomyosarcoma: A nomogram to better define patients who can be salvaged with further therapy. J. Clin. Oncol..

[60] Yang L., Takimoto T., Fujimoto J. (2014). Prognostic model for predicting overall survival in children and adolescents with rhabdomyosarcoma. BMC Cancer.

[61] Shen W., Sakamoto N., Yang L. (2014). Model to predict the survival benefit of radiation for patients with rhabdomyosarcoma after surgery: A population-based study. Int. J. Oncol..

[62] Gronchi A., Miah A.B., Dei Tos A.P., Abecassis N., Bajpai J., Bauer S., Biagini R., Bielack S., Blay J.Y., Stacchiotti S. (2021). ESMO Guidelines Committee, EURACAN and GENTURIS. Electronic address: Clinicalguidelines@esmo.org. Soft tissue and visceral sarcomas: ESMO-EURACAN-GENTURIS Clinical Practice Guidelines for diagnosis, treatment and follow-up^☆^. Ann. Oncol..

[63] Desmoid Tumor Working Group (2020). The management of desmoid tumours: A joint global consensus-based guideline approach for adult and paediatric patients. Eur. J. Cancer.

[64] Crago A.M., Denton B., Salas S., Dufresne A., Mezhir J.J., Hameed M., Gonen M., Singer S., Brennan M.F. (2013). A prognostic nomogram for prediction of recurrence in desmoid fibromatosis. Ann. Surg..

[65] Lazar A.J., Tuvin D., Hajibashi S., Habeeb S., Bolshakov S., Mayordomo-Aranda E., Warneke C.L., Lopez-Terrada D., Pollock R.E., Lev D. (2008). Specific mutations in the beta-catenin gene (CTNNB1) correlate with local recurrence in sporadic desmoid tumors. Am. J. Pathol..

[66] van Broekhoven D.L., Verhoef C., Grünhagen D.J., van Gorp J.M., den Bakker M.A., Hinrichs J.W., de Voijs C.M., van Dalen T. (2015). Prognostic value of CTNNB1 gene mutation in primary sporadic aggressive fibromatosis. Ann. Surg. Oncol..

[67] Tan P.H., Thike A.A., Tan W.J., Thu M.M., Busmanis I., Li H., Chay W.Y., Tan M.H., Phyllodes Tumour Network Singapore (2012). Predicting clinical behaviour of breast phyllodes tumours: A nomogram based on histological criteria and surgical margins. J. Clin. Pathol..

[68] Zivanovic O., Jacks L.M., Iasonos A., Leitao M.M., Soslow R.A., Veras E., Chi D.S., Abu-Rustum N.R., Barakat R.R., Brennan M.F. (2012). A nomogram to predict postresection 5-year overall survival for patients with uterine leiomyosarcoma. Cancer.

[69] Iasonos A., Keung E.Z., Zivanovic O., Mancari R., Peiretti M., Nucci M., George S., Colombo N., Carinelli S., Hensley M.L. (2013). External validation of a prognostic nomogram for overall survival in women with uterine leiomyosarcoma. Cancer.

[70] Tan B.Y., Acs G., Apple S.K., Badve S., Bleiweiss I.J., Brogi E., Calvo J.P., Dabbs D.J., Ellis I.O., Eusebi V. (2016). Phyllodes tumours of the breast: A consensus review. Histopathology.

[71] Chibon F., Lagarde P., Salas S., Pérot G., Brouste V., Tirode F., Lucchesi C., de Reynies A., Kauffmann A., Bui B. (2010). Validated prediction of clinical outcome in sarcomas and multiple types of cancer on the basis of a gene expression signature related to genome complexity. Nat. Med..

[72] Filleron T., Le Guellec S., Chevreau C., Cabarrou B., Lesluyes T., Lodin S., Massoubre A., Mounier M., Poublanc M., Chibon F. (2020). Value of peri-operative chemotherapy in patients with CINSARC high-risk localized grade 1 or 2 soft tissue sarcoma: Study protocol of the target selection phase III CHIC-STS trial. BMC Cancer.

[73] Frezza A.M., Stacchiotti S., Chibon F., Coindre J.M., Italiano A., Romagnosa C., Bagué S., Dei Tos A.P., Braglia L., Palmerini E. (2023). CINSARC in high-risk soft tissue sarcoma patients treated with neoadjuvant chemotherapy: Results from the ISG-STS 1001 study. Cancer Med..

[74] Foersch S., Eckstein M., Wagner D.C., Gach F., Woerl A.C., Geiger J., Glasner C., Schelbert S., Schulz S., Porubsky S. (2021). Deep learning for diagnosis and survival prediction in soft tissue sarcoma. Ann. Oncol..

[75] Crombé A., Roulleau-Dugage M., Italiano A. (2022). The diagnosis, classification, and treatment of sarcoma in this era of artificial intelligence and immunotherapy. Cancer Commun..

[76] Shahbaz S.K., Koushki K., Ayati S.H., Bland A.R., Bezsonov E.E., Sahebkar A. (2021). Inflammasomes and Colorectal Cancer. Cells.

